# A Sixty-Year Research and Development of Trichosanthin, a Ribosome-Inactivating Protein

**DOI:** 10.3390/toxins14030178

**Published:** 2022-02-27

**Authors:** Jia-Qi Lu, Kam-Bo Wong, Pang-Chui Shaw

**Affiliations:** 1 Centre for Protein Science and Crystallography, School of Life Sciences, The Chinese University of Hong Kong, Shatin, Hong Kong, China; lujq@link.cuhk.edu.hk (J.-Q.L.); kbwong@cuhk.edu.hk (K.-B.W.); 2 Li Dak Sum Yip Yio Chin R&D Centre for Chinese Medicine, The Chinese University of Hong Kong, Shatin, Hong Kong, China

**Keywords:** Tian Hua Fen, trichosanthin, abortifacient, rRNA N-glycosylase, anti-HIV, anti-cancer, immunotoxin

## Abstract

Tian Hua Fen, a herbal powder extract that contains trichosanthin (TCS), was used as an abortifacient in traditional Chinese medicine. In 1972, TCS was purified to alleviate the side effects. Because of its clinical applications, TCS became one of the most active research areas in the 1960s to the 1980s in China. These include obtaining the sequence information in the 1980s and the crystal structure in 1995. The replication block of TCS on human immunodeficiency virus in lymphocytes and macrophages was found in 1989 and started a new chapter of its development. Clinical studies were subsequently conducted. TCS was also found to have the potential for gastric and colorectal cancer treatment. Studies on its mechanism showed TCS acts as an rRNA *N*-glycosylase (EC 3.2.2.22) by hydrolyzing and depurinating A-4324 in α-sarcin/ricin loop on 28S rRNA of rat ribosome. Its interaction with acidic ribosomal stalk proteins was revealed in 2007, and its trafficking in mammalian cells was elucidated in the 2000s. The adverse drug reactions, such as inducing immune responses, short plasma half-life, and non-specificity, somehow became the obstacles to its usage. Immunotoxins, sequence modification, or coupling with polyethylene glycerol and dextran were developed to improve the pharmacological properties. TCS has nicely shown the scientific basis of traditional Chinese medicine and how its research and development have expanded the knowledge and applications of ribosome-inactivating proteins.

## 1. Introduction

Trichosanthin (TCS) is generated from the extract of the root tuber of *Trichosanthes kirilowii* (Gua Lou) from the family of Cucurbitaceae [1]. The root tuber has long been used in traditional Chinese medicine (TCM) as an abortifacient called Tian Hua Fen [2]. The traditional medical book *Qianjin Yifang* by Sun Simiao was the first recording of Tian Hua Fen in the 7th century. The others, including *Taiping Royal Prescriptions* by Wang Huaiyin and *Compendium of Materia Medica* by Li Shizhen have also recorded it in prescriptions to deal with abortion, abnormal menstruation, and retained placenta [3].

**Figure 1:**
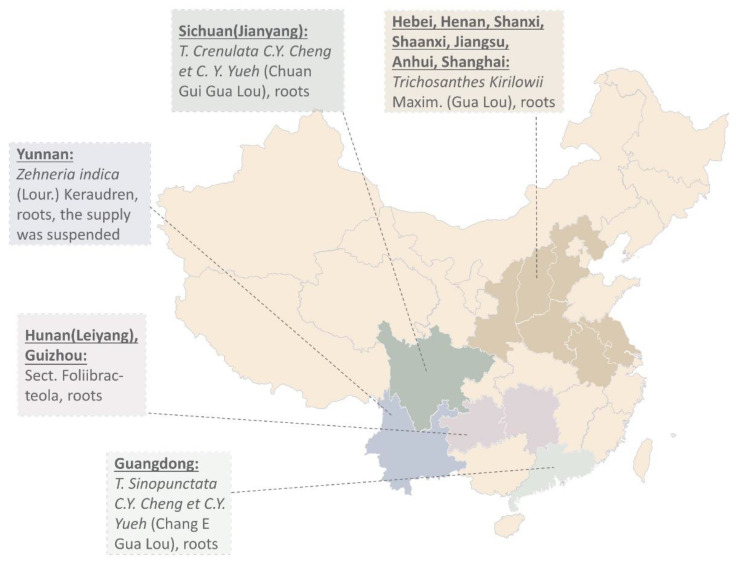
The sources of commercial Tian Hua Fen in China in the 1980s [3,7]. Researchers used the microscopic identification method to identify and authenticate commercial Tian Hua Fen in 21 provinces in China in the 1980s. The corresponding species names of their origins are shown on the map.

Ancient records show different plant resources of Tian Hua Fen. Records in *Chongxiu Zhenghe Jingshi Zhenglei Beiyong Bencao* show two Gua Lou from Hengzhou and Junzhou [4]. The latter was used as Tian Hua Fen for abortion. Le et al. offer over 30 types of Gua Lou used in China [5,6]. Some of them were misused. One of them is the root of *T. cucumeroides* (Ser.) Maxim (Wang Gua). Others such as *T. cavaleriei* Levl. (Chang Mao Gua) and *T. hupehensis.* C. Y. Cheng et C. Y. Yueh (Hu Bei Gua Lou) have more side effects such as nausea and vomiting. Gua Lou that produces TCS mainly comes from Boxian in Anhui, Anyang in Henan, Pudong in Shanghai, and Nantong in Jiangsu [3] (Figure 1). Harvest of Gua Lou was usually carried out in late autumn to early spring. The root was peeled, cut into slices, and soaked in water for 5 days. The water was changed every day. Tian Hua Fen was then extracted from the clarified and sun-dried white powder.

Monographs were published in 1990 [8] and 2000 [3] reviewing the efforts on TCS research in China and over the world. As with Artemisinin, the study on Tian Hua Fen represents drug development from traditional Chinese medicine (TCM) and illustrates the scientific basis of TCM. In this review, we look back to the sixty-year research and development of TCS as an abortifacient, rRNA N-glycosylase, anti-HIV and anti-cancer agents to reveal the fascination of TCS.

## 2. Trichosanthin as an Abortifacient

### 2.1. From Ancient Prescriptions to Crystallized Protein Powder

Because of the lack of western drugs, Tian Hua Fen was used extensively in China for inducing abortion in the 1960s [2]. The prescription contains Tian Hua Fen, dried sterile fruit of *Gleditsia sinensis* (Ya Zao), *Asarum sieboldii* (Xi Xin), the root *of Euphorbia fischeriana* (Lang Du) or with three more herbs, including *Angelica dahurica* (Bai Zhi), the root of *Kaempferia galanga L.* (Shan Nai), and the root of *Nardostachys chinensis Batal* (Gan Song).

The herbs were crushed to make a liquid extract as a medicated vaginal suppository (4) to induce abortion. However, because of severe adverse drug reactions (ADR) such as fever, tetter, and abdominal pain, studies on revising the prescription were carried out. In 1965, researchers in Wuhan and Nanjing, China, conducted thorough studies on the chemical, pharmacology, and clinical characteristics of the prescriptions to improve its pharmacological performance. The original prescriptions were simplified to contain only Tian Hua Fen and Ya Zao, called “Tian Zao He Ji” in powder form as capsules in the late 1960s [3,9,10]. Tian Hua Fen was also precipitated with methanol and ethanol and assembled with Ya Zao as capsules called “Tian Ya San” [3].

However, ADR was still a concern in its application. In 1966, researchers in Shanghai further isolated and purified components in Ya Zao and Tian Hua Fen. One death report in clinical application in 1969 alerted researchers again on the impact of ADR. The researchers showed that only Tian Hua Fen could induce abortion in pregnant mice, while Ya Zao only causes muscle festering. This suggests Tian Hua Fen was the component for abortifacient, while Ya Zao was only an enhancer, profiting from its surfactant characteristic [9]. The effects of Tian Hua Fen in ectopic pregnancy and chorionic epithelioma were also reported [2,11,12].

**Figure 2:**
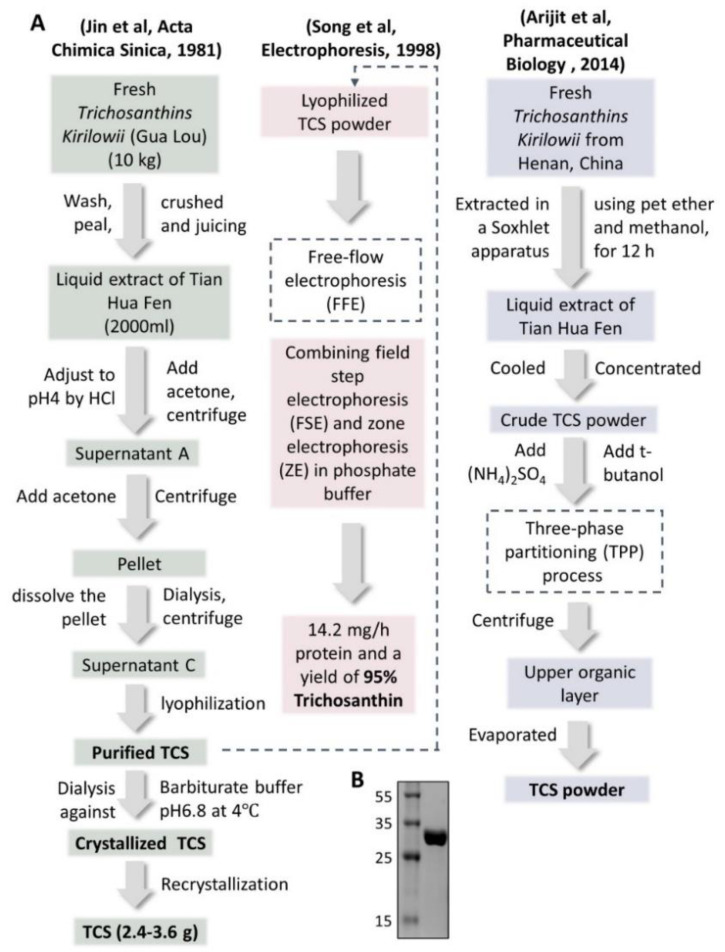
Extraction and purification steps of crystalized/purified TCS powder. (**A**) Three reported extraction and purification steps of TCS from *Trichosanthes kirilowii* are listed. Recrystallization [12], FFE (free-flow electrophoresis) [13] and TPP (three-phase partitioning) [14] processes were used. TCS: trichosanthin. (**B**) 15% SDS-PAGE gel photo of purified TCS in our lab.

Tian Hua Fen itself was precipitated and made as injections, first used in 1972. The dosage was 5 mg per dose. It was then developed as more purified injections in 1975 with only 2 mg per dose. In 1982, the precipitated powder was further crystallized in barbitone buffer and came out as the single protein powder of trichosanthin (TCS) [10]. The dosage of these developed injections was further lower to 1.2 mg per dose [13], which won the second prize of the National Invention Award. The flowchart of three representative isolation and purification methods of TCS from *Trichosanthes Kirilowii* in different ways are shown in Figure 2, the SDS-PAGE gel photo of purified TCS is also demonstrated (Figure 2B).

The clinical application was synergistically developed with its mechanism study. The mechanism on its midterm abortion and anti-early pregnancy effects were reported relying on the damage of syncytiotrophoblast cells [15] and the selective damage on chorionic tissue [16].

### 2.2. Clinical Application of Tian Hua Fen or TCS as an Abortifacient

The clinical studies of Tian Hua Fen and crystallized TCS powder were firstly on midterm abortion. They then expanded to anti-early pregnancy, ectopic pregnancy, molar pregnancy, and chorionic epithelioma. Tian Hua Fen was a topical medication for the vagina when used as a prescription for TCM. Then it was used for muscle, amniotic cavity, cervix uteri, or intrauterine injections with a simplified prescription and further purification (Table 1).

For midterm abortion, gravidas in 12–24 weeks pregnancy were treated with injections after a skin test [2]. The abortion occurred within seven days. ADR of Tian Hua Fen includes whole-body flu-like response (fever, headache, arthralgia, pharyngalgia, etc.), tetter, pain in the injection area, gingival and nose bleeding, abdominal pain, infection, severe allergic reactions, and massive hemorrhage [29]. To avoid these ADR, dexamethasone was used to alleviate side effects and prevent anaphylaxis [16]. According to a long-term follow-up on 528 patients, the midterm abortion by Tian Hua Fen did not show a notable impact on their health, menstruation, and fertility, nor on the development and intelligence of next generations.

Tian Hua Fen was also used in stopping early pregnancy [30]. As with midterm abortion, gravidas, within 12 weeks of pregnancy, were treated with injections after a skin test. The abortion occurs within two weeks. Flu-like symptoms were also the principal ADR. Nowadays, medical abortions mainly rely on hormones such as mifepristone and misoprostol, which show higher success rates and fewer side effects [31].

### 2.3. Protein and DNA Sequences of TCS

The primary structure (protein sequence) of TCS was first reported in the Symposium on the Organic Chemistry of Medicinal Natural Products held by the International Union of Pure and Applied Chemistry (IUPAC) in Shanghai in 1985 [32]. Wang et al. reported the TCS consists of 233 amino acids. In 1990, Collins et al. reported a slightly different sequence that consists of 247 (246) amino acids [33], which was later corroborated by Wang et al. [34]. The C-terminus of TCS has microheterogeneity, proved by the ESI-MS method [3]. Two peptides from the C-terminal of TCS were found ending at Ala and Met [35]. Multiple sequence alignment of TCS with other RIPs such as Karasurin, luffin, α-MMC, and PAP shows several conserved amino acids.

**Figure 3:**
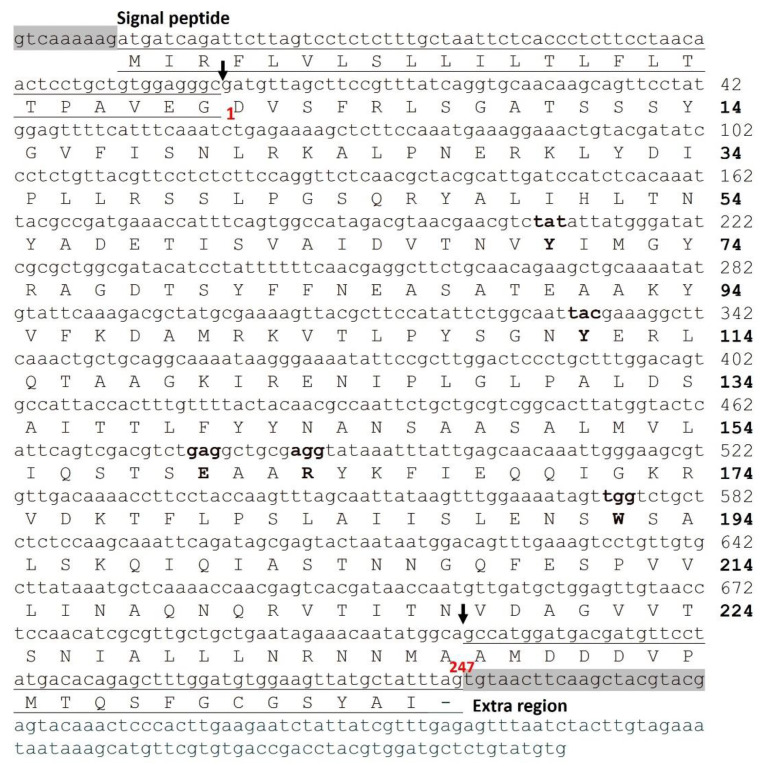
The DNA and protein sequences of TCS. The primary and nucleotide sequences of TCS (UniProt accession: Q6BBQ4; GenBank: M34858.1). The amino acids in the active centre are marked in bold. The signal peptide (−23–−1 aa) at *N*-termini and extra region (248–266 aa) at C-termini are underlined.

Our group prepared the cDNA library of the root tuber of *T. kirilowii*, and cloned and sequenced the cDNA of TCS [36]. Recombinant TCS was then synthesized in *E. coli* [37]. This opened the door for using site-directed mutagenesis to study the molecular mechanism of TCS. Figure 3 shows the primary and nucleotide sequences of TCS. The multiple sequence alignments of TCS and other RIPs are also presented (Supplementary Figure S1). The mature TCS protein has 247 amino acids (aa), while the Pre-TCS contains a signal peptide (UniProt entry Q8LPV7/Q41611, 270 aa) and an extra region at C-terminus (UniProt entry Q41216/ Q84SV8, 289 aa) (Figure 3).

## 3. Trichosanthin as an *rRNA N-glycosylase*

### 3.1. Mechanism of TCS as an rRNA N-glycosylase

Trichosanthin was shown to be an rRNA *N*-glycosylase (EC 3.2.2.22) in 1992 and was classified as a ribosome-inactivating protein (RIP) [38]. As with ricin [39], TCS catalyzes the hydrolysis of the *N*-glycosidic bond of A-4324 on the conserved sarcin–ricin loop (SRL) on the 28S rRNA of rat ribosome [38].

RIPs have substrate specificity. Taking the ricin A chain (RTA) as an example, RTA cannot depurinate intact ribosomes from *E. coli* but can react on naked 23S rRNA. Sequences of the 28S and 23S rRNA in α-SRL are highly conserved. Endo et al. showed that ricin can retain its specificity mimic α-SRL on 35-mer synthetic oligoribonucleotide [40], which confirmed the importance of this loop on the depurination activity of RIPs.

Depurination of the SRL inhibited the binding of the elongation factors to the ribosomes and suppressed the activation of GTPase [41,42,43]. In 2010, Voorhees et al. reported that SRL is located next to the translation GTPase binding site and directly modulates hydrolysis activation [44]. SRL exhibits conformational changes after depurination by RIPs, inhibiting the activation of GTP hydrolysis.

### 3.2. Mechanism of TCS as Revealed by Structural Studies

In the 1990s, crystal structures of TCS and other RIPs together with their substrates or analogues, including Formycin 5′-monophosphate (FMP), adenyl(3’→5’)guanosine (ApG), ATP, adenosine (ADO), cytine monophosphate (CMP), tubercidin (Tub), and NADPH, were studied [45,46,47,48], providing the structural information of the catalytic centre and the foundation of mechanism study [49,50]. The detailed geometries of the active centers of TCS and other RIPs such as α-momorcharin (MMC) are described and compared [46]. Only adenine molecules remained in the active centre in most crystal structures with the above substrates or analogues, suggesting the depurination reactions were already completed.

**Figure 4:**
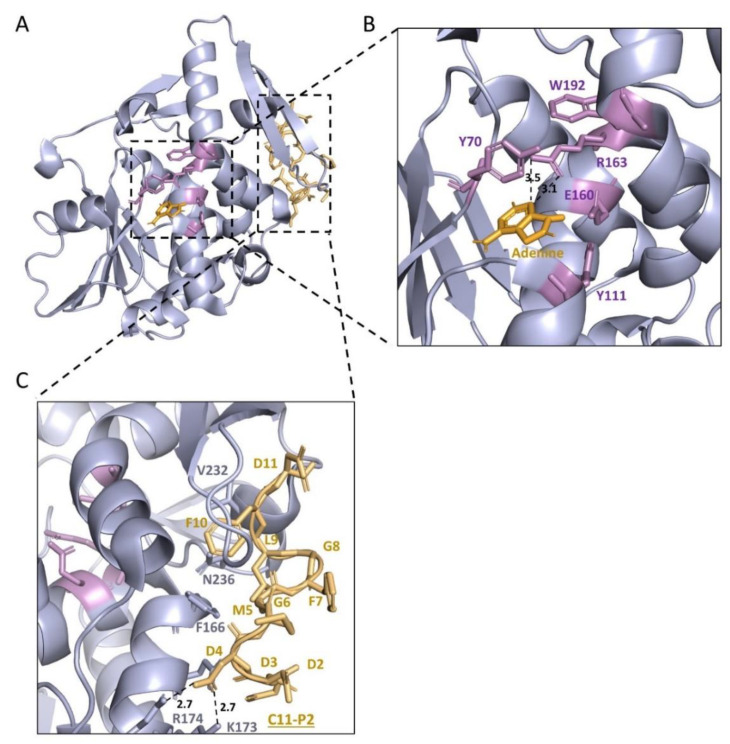
Structure basis of TCS as an rRNA *N*-glycosylase (EC 3.2.2.22). The structure of TCS with P2 (PDB code: 2JDL) was combined with the structure of TCS and adenine (PDB code: 1MRJ). (**A**) The protein structure of TCS is shown in a light blue image. (**B**) The active centre of TCS (Y70, Y111, E160, R163, W192) is shown in violet sticks. The active centre of TCS is conserved in RIPs. Adenine molecule is shown as bright orange sticks. Dash lines indicate hydrogen bonds between adenine molecule and TCS, and related distances (Å) are marked. The two tyrosine residues are stacking with adenine. (**C**) Interaction of TCS and C11-P2. C11-P2 (DDDMGFGLFD) is shown in light orange. C11-P2 interacts with the C-termini of TCS. Hydrogen bonds between C11-P2 peptide and TCS are shown in dash lines, and related distances (Å) are marked. RIPs: Ribosome-inactivating protein; TCS: trichosanthin; C11-P2: 11 amino acids of P2 protein in the C-termini (only ten amino acids are shown in the figure).

The structure of TCS (Figure 4a) consists of a large N-terminal domain with six alpha-helices, a six-stranded sheet, an antiparallel beta-sheet, and a small C-terminal domain with the largest distinct bent alpha-helix (Figure 4a) [51]. There is a cleft between which is also located the conserved domain of RIPs (Supplementary Figure S1). This cleft was conjectured as the active cleft, which was confirmed by mutagenesis studies in the 1990s [52,53,54]. In the active center, Tyr70, Tyr111, Glu160, Arg163, and Phe192 are important in their activity and interaction with adenine (Figure 4b) [50]. Ile71 and Gly109 form hydrogen bonds with N(1) and N(6) of the adenine molecule in the active site [54,55]. The structural comparison of TCS with other RIPs was conducted recently [56].

In the proposed mechanism [1,36,37], the adenine ring of A-4324 of 28S rRNA is protonated by Arg 163. This attracts the electron flow from the ribose oxygen to the ring that breaks the N-glycosidic bond. The resulting oxocarbenium intermediate is stabilized by Glu 160 and is used to stabilize the oxocarbenium, further enhancing reaction rates. The regeneration of the rRNA *N*-glycosylase is contributed from the subsequent nucleophilic attack of C-1 of the water molecule to the ribose.

### 3.3. Interaction of TCS with the Ribosome and the Recruitment by Ribosomal P-Protein

TCS interacts with ribosomal stalk P-proteins and L10a [57,58]. Ribosomal P-proteins, including uL10 (previously known as P0 [59]), two P1, and P2, have highly conserved acidic sequences in the C-termini [58]. The two dimers of the stalk are arranged in parallel and interact respectively with uL10 [60]. Through chemical shift perturbation and mutagenesis analyses, the interaction with acidic ribosomal stalk proteins was found to help TCS to locate its RNA substrate [61]. This depurinated ribosome could not bind the elongation factors, thereby inhibiting the protein synthesis [61]. Ribosomes with depurinated SRL can also be a signal for halting the cell cycle [62].

In 2009, the co-crystallization of this C-terminal 11 amino acids peptide (C11-P) and TCS revealed the binding mode (Figure 4c). The ribosomal proteins interact with TCS at antiparallel β-sheet 9 and 10 with charge–charge interaction and hydrophobic bonds [63]. In 2013, Lee et al. showed the flexible C-terminal tails of P1/P2 heterodimer could reach up to ~12.5 nm from the ribosomal stalk to recruit elongation factors or RIPs to the ribosomes [64,65,66]. The multiplication of P-proteins was found to contribute to the fidelity of translation [67], and multiple P-proteins also accelerated the interaction of RIPs and ribosome [68]. In 2017, Grela et al. showed functional non-equivalence of the individual P-protein CTDs [69]. In 2021, Horbowicz-Drożdżal et al. revealed that phosphorylation of the conserved C-terminal domain of ribosomal P-proteins impairs the interaction with plant toxins [70]. The study of how TCS interacts with ribosomes became a model for similar work in other RIPs [71].

## 4. Trichosanthin as an Anti-HIV Agent

### 4.1. Anti-HIV Activity of TCS and Its Related Mechanism

In 1989, McGrath et al. first reported the inhibitory activity of TCS on HIV replication in HIV-sensitive T-lymphoblastoid cells (VB cell line) [72,73]. Highly purified, formulated preparation of TCS, GLQ223 selectively reduced HIV p24 level. In 1991, Ferrari et al. used HIV-infected MT4, H9, and CEM-ss cells to evaluate the anti-HIV activity of TCS [74]. TCS inhibited syncytium formation in injected H9 cells and uninfected Sup-T1 cells from 0.5 to 4 µg/mL. The HIV replication in infected H9 and CEL-ss cells can also be inhibited at 0.25 µg/mL. A similar anti-HIV activity was also detected in primary cultures of monocyte/macrophages chronically infected with HIV [73].

The mechanism of the anti-HIV activity of TCS is not entirely clear. However, its rRNA *N*-glycosylase activity and enhancement of chemokines to stimulate chemotaxis and activation of pertussis toxin-sensitive G protein were considered the most important [75]. In recent decades, RIPs were reported to affect various processes in the life cycle of HIV, such as reverse transcription, integration, replication, and assembly [76].

Temporary interaction of TCS with HIV-1 long-terminal repeats (LTRs) can affect the HIV integration step [77]. TCS-enriched virions have HIV-1 scaffold protein Gag and lipid raft membrane, which can inhibit the infection ability of HIV [78]. Nicking endonuclease activity and DNase-like activity of TCS on DNA are also possible mechanisms [79]. Wang et al. raised that the anti-HIV activity of TCS may also be indispensable with its RIPs activity. The exceptions were TCS variants TCS-C19aa (19 amino acids C-terminal extension) and TCS-KDEL (signal sequence at C-terminal) [80]. These two variants retained all ribosome-inactivating (RI) activity but subsequently lost most of the anti-HIV-1 activity. The anti-HIV activity of TCS was also reported related to the MAPK signal pathway [81,82].

Lendray et al. in 1991 reported the treatment of GLQ223 on Simian immunodeficiency virus (SIV)-infected rhesus macaques [3]. Three SIV-infected rhesus macaques with detectable SIV, systemic lymphadenopathy, and decreased CD4/CD8 were treated with TCS at 200 µg/kg 1–2 times a week by intravenous infusion (i.v.). ADR included flushing of the face, tremor, less physical activity and appetite, reversible and transient decreases of serum glutamic oxaloacetic transaminase (SGOT), serum glutamic pyruvic transaminase (SGPT), and lactate dehydrogenase (LDH). The experiment was suspended for one rhesus macaque because of the allergy. The second developed severe SIV diseases, and the third showed significantly longer survival.

### 4.2. Clinical Study of TCS on AIDS Patients

In the 1990s, after McGrath et al. reported the anti-HIV potential of TCS, the Food and Drug Administration (FDA) permitted the clinical study of TCS on HIV using purified and formulated preparation GLQ223 [18,83]. Unofficial clinical trials were also conducted using Shanghai Jinshan Pharmaceutical Factory injections. In addition, more than 1000 patients also tried treatment with TCS voluntarily in China.

For the Phase I clinical study, increasing dosages of TCS were used to measure the kinetic parameters such as biological half-life time (t1/2), serum clearance rate, distribution, and ADR observation [18,20,23,83]. For medium or lower dosages (1, 8 µg/kg), the dosages were well tolerated. For higher dosages (16, 24, 36 µg/kg), 12 patients complained of flu-like symptoms. One patient showed severe neurological symptoms with semiconscious, prostration, and internuclear ophthalmoplegia [23]. Higher dosages and longer i.v. time were then carried out in trials by Ciesielka et al., which showed a tolerance to 500 µg/kg in patients with TCS antibodies developed [3].

Phase II clinical trials were conducted in azidothymidine (AZT)-invalid HIV patients [19,84,85,86,87]. Results showed that combining TCS and other treatments such as AZT was a promising method [19,87]. Byers et al. in 1991 reported a clinical study with 112 patients using the dosage of 1.2 mg/week for two weeks and following with one injection of TCS per month in combination with AZT and Didanosine (DDI). Results showed TCS could be used for synergistic treatment and reverse the CD4 T cells decrease [19,87]. However, a higher dosage was needed to further inhibit HIV replication [3]. The following phase II study of the addition of trichosanthin to zidovudine in patients with HIV disease and failing antiretroviral agents from the same research group [26] showed similar results. Nevertheless, none of the above studies were double-blind controlled, affecting the reliability. PEGylation modification [88] and immunotoxin (ITs) [89] were made to improve the pharmacological properties of TCS, as elaborated in the latter section (Section 6).

The clinical studies of TCS are listed in Table 1. TCS showed potential in HIV treatment. However, the side effects, including flu-like symptoms and reversible mental status changes, including dementia and even coma [76], hindered its further applications. Currently, cocktails-high active antiretroviral therapy is used for most AIDS treatments [90].

## 5. Trichosanthin as an Anti-Cancer Agent

### 5.1. The Entering of TCS into Cells

Trafficking of RIPs into the cells and interacting with ribosomes are essential steps for their cytotoxicity [91]. TCS is a Type I RIP, consisting of a single chain polypeptide with enzymatic activity. Type II RIPs such as ricin and abrin have a lectin binding B chain linked with the enzymatic A chain by disulfate bonds. In 1976, Sandvig et al. first reported the kinetics parameters of binding the lectin B chain of type II RIPs ricin and abrin to the human surface receptors [92]. In recent decades, lots of studies have revealed the mechanism [93].

Without the assistance of the lectin B chain, TCS uses receptor-mediated endocytosis and non-specific entry to enter the cells [51]. In 2003, Zhang et al. reported that the seven amino acids in the C-termini mimic the function of the lectin binding domain [94]. Under acidic conditions, it interacts and inserts the toxin into the phospholipid bilayer. This facilitates its translocation to the cytosol. In the 2000s, researchers showed that carriers and receptors such as low-density lipoprotein receptor-related protein (LRP), megalin, and clathrin-coated vesicles interact with TCS to form endosomes. Lysosome then helps digest and facilitate the relocation [94,95,96].

### 5.2. Anti-Cancer Properties of TCS

TCS has potential anti-cancer activities on tumors in the female reproductive system (breast cancer, cervical cancer, and choriocarcinoma), immune system, digestive system (colon cancer, hepatoma, and gastric cancer), blood system, respiratory system, and others [51]. The start of anti-cancer studies of TCS began on gastric and colorectal cancers [3]. In 1993, Wu et al. reported the cytotoxicity of TCS on gastric (MKN-45, SGC-7901, MKN-28) and colorectal cancer (SW-1116, SGC-7402, and ras-positive Wef) cell lines in vitro [97]. In 1995, Zheng et al. showed TCS has selective cytotoxicity on leukemia–lymphoma cells in vitro, which also raised the potential of its treatment on some lymphomas and leukemia [98].

In the SGC-7901-based gastric cancer transplant tumor nude mouse model, TCS also suppressed tumor growth in vivo with an effective suppression dose of 0.5 mg/kg, far lower than the median lethal dose (LD50) 13.4 mg/kg. Combined administration of TCS with fluorouracil (5-FU), mitomycin (MMC), and interferon α-2b showed higher cytotoxicity on gastric and colorectal cancer cell lines. Takemoto et al. in 1998 reported the anti-tumor activity of the N-terminal sequence of trichosanthin in vivo [99].

The use of TCS to combat cancer is an active research area. Chen et al. showed that TCS inhibits the proliferation, migration, and epithelial–mesenchymal transition of human cervical cancer cells. These were considered mediated by inhibiting the STAT5/C-myc signaling pathway [100]. Smac was shown as another pathway in the anti-cancer activity of TCS in CaSki cervical cancer cells [101]. Zhu et al. showed that regulating oxidative stress-induced apoptosis is one of the TCS mechanisms that inhibit cervical cancer [102]. Tang et al. developed a co-delivery of TCS and Albendazole by the nano-self-assembly system, overcoming multidrug-resistance and metastasis [103]. TCS was found to have the potential to increase the sensitivity of non-small cell lung cancer (NSCLC) TRAIL-resistance cells [104]. The proposed mechanisms of anti-cancer and TCS-induced apoptosis pathways were reviewed by Shi et al. [51]. All these efforts may be favorable for its clinical applications.

## 6. Engineering and TCS-Based Immunotoxins

The engineering and modification studies of RIPs include these aspects: (a) immunotoxins (ITs) made to improve pharmacological performance; (b) engineering of signal sequences to improve specificity; (c) coupling with PEG or dextran to increase the plasma half-life. A pharmacokinetic study of TCS in mice showed that the kidney excreted TCS through urine; the half-life was 8.4–12.7 min. Ko et al. reported coupling with dextran could prolong plasma half-life and minimize renal loss [105]. TCS coupling with PEG5k on Q219C/K173C/S7C showed no significant changes in RIPs activity and cytotoxicity, while the mean residence time and binding affinity of an IgE monoclonal antibody (TE1) to TCS were improved [106]. ITs were also made to improve the specificity of other RIPs. For example, ricin A chain (RTA) and pokeweed antiviral protein (PAP) were linked with HIV envelope glycoprotein and surface antigens for targeting [107,108,109].

Our group also tried to improve the specificity of RIPs by linking RIPs with HIV-1 protease recognition sequences to the C-termini or the internal inactivation region of Maize RIP variants or RTA [110,111,112]. A switch on a mechanism based on the recognition sequences was proposed to realize higher specificity to the HIV-infected cells instead of normal cells. Subsequently, an in vivo study on Simian immunodeficiency virus (SIV)-infected rhesus macaques showed that recombinant active Maize RIP had better-reduced plasma viral burden transiently [112].

Immunotoxins usually consist of a vector part for interaction and a toxin part as the “bullet”. The vectors need high affinity, specificity, high stability, and long half-life to the target. Clusters of differentiation (CD) on hematological cells’ surfaces are among the most popular used ITs targets. Monoclonal antibodies (mAb) against various cancer antigens, hormones and growth factors, and cytokines were also used [3,107]. The “bullet” of immunotoxins are usually toxins from animals (diphtheria, pseudomonas aeruginosa exotoxin) or plants (ricin, trichosanthin, abrin). The target toxin can be expressed together with the vector or conjugated chemically [113].

In 1987, Wang et al. in China reported using TCS conjugated with monoclonal antibody (mAb) on liver cancer [114]. In 1988, Pierre et al. made immunotoxin based on trichokirin, extracted from *T. Kirilowii,* and has high homology with TCS [115]. A cleavable dimethyl 3,3′-dithiobispropionimidate cross-linking agent was used to link trichokirin with a monoclonal antibody against Thy 1.2 antigen. The activity of TCS-based ITs was slightly impaired, but the clearance rate was longer compared with ITs of RTA [116]. ITs based on TCS were potent and caused a selective and specific depletion of cholinergic neurons in neostriatum, which performed better than a commercially available immunotoxin containing saporin [117]. These showed that TCS might be a good candidate in immunotoxin development.

In the 1990s, researchers reported the use of ITs of TCS with Hepama-1 (human hepatoma) [89], CMU15A (Lung cancer antigen) [118], anti-p75-anti-mouse IgG (p75: nerve growth factor) [117], Ng76 (Melanoma) [119], and EGF (hepatocellular carcinoma) [120,121,122] (Table 2). A review on the use of RIPs as immunotoxins is available [76].

## 7. Conclusion and Perspectives

Various historic medicines are still sparkling with the modification of advanced technology. Insulin was isolated from the internal secretion of the pancreas by Banting and Macleod in the 1920s [128]. It was then applied to commercialization and bench-to-bedside application for diabetes. Further optimizations of insulin in the following decades were conducted to improve its performance [129]. Recombinant proteins were cloned and used for expression in the 1980s [128,130]. Similar to insulin, TCS is also a natural product. Studies on TCS also formed a history of intelligent collaboration, ceaseless renovation, and bench-to-bedside research.

For the clinical applications of TCS, there are three active areas of work. Before the 1980s, it was abortion. In recent decades, anti-HIV and anti-cancer have become more active. Clinical requirements first motivated the application of TCS as an abortifacient. The ADRs reflected from the clinical application then drove the modification and engineering of TCS. New biological activities inspired novel applications. Its molecular and structural basis as rRNA *N*-glycosylase (EC 3.2.2.22) supported its engineering for improvement. These made TCS become a showcase of bench-to-bedside translation and drug development from TCM.

As well as clinical applications, TCS has been shown to inhibit plant viruses. Local lesion formation by turnip mosaic virus (TuMV) in leaves of *Nicotiana tabacum* was inhibited by exogenous application of recombinant TCS. It also caused a delay in the development of mosaic symptoms by TuMV in *Brassica parachinensis* [131]. The transgenic plant TP3, TP11 with the gene of TCS have shown virus resistance on tobacco mosaic virus (TMV) and cucumber mosaic virus (CMV) [132,133].

After TCS, various other RIPs were studied. The distribution of RIPs was also expanded from plants to bacteria [134], animals [135], fungi [136,137], and even mosquitos [138]. A number of these have protein sequences different from the classical RIPs. These new RIPs reveal new mechanisms based on different catalytic centers. An example is lyophyllin from *Lyophyllum shimeji*. This RIP is a member of the peptidase M35 superfamily but has potent rRNA *N*-glycosylase activity [139].

For pharmaceutical uses, immunogenicity, short plasma half-life, and non-selective cytotoxicity were the major issues to be overcome. Its immunogenicity may cause reduced efficacy through chronic exposure. As well as coupling with polyethylene glycerol and dextran, deimmunization by depleting T-cell epitopes on the protein can be a good way to reduce immunogenicity in designing ITs [140]. Recent studies showed that the clearance of partially unfolded or oxidized proteins by non-target cells such as hepatic Kupffer cells may also affect the cytotoxicity and tolerance of TCS [141,142,143]. Strategies to improve specificity, such as targeting the protein delivery system [144], show potential to overcome this problem.

There are still many opportunities in the research and development of TCS. New techniques in nanoparticles, co-delivery systems, and tumor-targeting protein delivery systems [103,145] may improve the specificity and reduce the side effects. TCS may be a component of a drug cocktail or combination therapy to increase the efficacy [104,146]. The penetrating peptide (CPP)-fused recombinant TCS toxin conjugated to lactoferrin (LF) has the potential to penetrate the blood–brain barrier (BBB) [146]. A cancer vaccination platform based on TCS was also implemented in 2021 [147], which is a promising tool for cancer immunotherapy. In basic science, studies on the TCS-ribosome complex by Cryo-EM will reveal the detail of interaction.

In conclusion, TCS has emerged from an abortifacient to an anti-HIV and anti-cancer agent. Over the years, modifications have been carried out to improve pharmacological performance, and mechanism studies have been conducted to understand the enzymatic reaction. TCS has become a showcase for the mechanism of function and applied values of this class of proteins. The research and development on TCS have also revealed the hidden treasure in Chinese medicine.

## Figures and Tables

**Table 1:** Clinical studies using Trichosanthin (TCS).

Time	Country/City	Agent	Target Disease	Effect	ADR ^1^	References
1989 ^2^	CA, US ^3^	GLQ223 ^4^	AIDS	-	-	[17]
1990	CA, US	GLQ223	AIDS	No consistent or sustained changes ^5^	No significant toxicity, except one with a severe neurological ADR.	[18]
1990	Nottingham, UK; CA, US; FL, US ^6^	GLQ223 (Phase I/II)	AIDS	Serum p24 antigen decreased after one month in 10–18 patients; one converted to negative.	Severe fatigue and myalgias; dementia or even coma in two patients; one death	[19]
1990	Shanghai, China	TCS^7^	Abortion (10–14 weeks)	High success rate, better in cervical injections	Increased body temperature; pain at the injection site; alleviated by dexamethasone	[11]
1991	CA, US; Pavia, Italy	GLQ223	AIDS	Pharmacokinetic study; predictability of elimination and distribution among species	-	[20]
1991	Shanghai, China	TCS with dexamethasone	Midterm abortion and anti-early pregnancy	High success rate (>83%)	Flu-like syndrome; can be alleviated with dexamethasone	[16]
1992	Shanghai, China	TCS	AIDS	Reduced p24 antigen, increased CD4 cells	Flu-like syndrome; pain and erythema at the injection sites	[21]
1993	Nottingham, UK; CA, US; MD, US ^8^	GLQ223	AIDS	-	Two patients developed coma; multifocal neurological deficits after treatment.	[22]
1993	CA, US; WA, US ^9^	GLQ223	AIDS	For patients who received 36 and 50 μg/kg, an increase in CD4+ and CD8+ T cells was sustained; Beta-Microglobulin levels increased during the infusion.	Flu-like syndrome with muscle; joint aches; increase in creatinine kinase levels	[23]
1994	Shanghai, China	TCS with dexamethasone	Abortion	High success rate (100%)	Flu-like syndrome; alleviated with dexamethasone	[24]
1994	Shanghai, China	TCS	Abortion	-	TCS may have a transient effect on the myocardium	[25]
1994	Nottingham, UK	GLQ223 combined with zidovudine	AIDS	Significant increase in CD4^+^ cell levels	Myalgias, fevers, mild elevation in liver function tests; mild-moderate anaphylactic reactions; two with mental status changes	[26]
2000	Shanghai, China	TCS	Tubal pregnancy	92% success rate	Flu-like syndrome	[27]
2001	Shanghai, China	TCS	Midterm abortion	High success rate (98%)	Flu-like syndrome	[28]

^1^ ADR: adverse drug reactions. ^2^ 1989: a clinical trial in 1989 was halted in August by FDA, and then resumed with full FDA approval with a new design to evaluate the safety and effectiveness of GLQ223 over a longer term in San Francisco, Los Angeles, Miami, and Florida. ^3^ CA, US: California, USA. ^4^ GLQ223: also called Compound Q, a highly purified, formulated form of TCS. ^5^ Serum concentration of GLQ223 was around effective concentration in vitro but has not been maintained for a sufficient duration. ^6^ Fl, US: Florida, USA. ^7^ TCS: trichosanthin, purified protein from *Trichosanthes kirilowii*. ^8^ MD, US: Maryland, USA. ^9^ WA, US: Washington, DC, USA.

**Table 2:** Immunotoxins (ITs) based on TCS.

Time	Immunotoxin	Target Antigen	Disease	Effect	References
1991	TCS-Hepama-1	Hepatoma-associated antigen	Hepatoma	Potent and specific antihepatoma agents; might have considerable potential in hepatoma therapy.	[89,123]
1992	B_3_-IgG-TCS	CEA ^1^-MAb antigen	Colorectal cancer; Cervical cancer	Anti-tumor activity in vitro and in vivo	[124]
1993	TCS-Hepama-1-gold	Hepatoma-associated antigen and gold	Hepatoma	TCS-Hepama-1-gold particles are bound to the microvilli of human liver carcinoma cells and can be inhibited competitively by pretreatment with Hepama-1 for an hour.	[125]
1995	CMU15—TCS	Lung cancer antigen	Lung cancer	Significant tumor-inhibited effect in vivo, inhibition rate of tumor growth reaching 76% in the group of peritoneal injection and 99.4% in the group of intra-tumor injection without apparent toxic effect to host mice	[118,126]
1996	TCS-Ng76	Melanoma antigen	Melanoma	The affinity gel may be used to purify different TCS-composed immunotoxins.	[119]
1997	H-2 Ag-TCS	Mouse major histocompatibility complex antigens (H-2 Ag)	Rat cardiac xenografts	The proliferation of recipient immune cells pretreated with conjugate H-2Ag-TCS was inhibited. H-2 Ag-TCS significantly prolonged the cardiac survival time	[127]
1999	p75-TCS (anti-p75 anti-mouse IgG-TCS)	Rat nerve growth factor (NGF) receptor (p75 receptor) (p75NTR)	Immuno-lesioning (cholinergic basal forebrain neurons)	Potent and caused a selective and specific depletion of cholinergic neurons in the neostriatum	[117]
2010	EGF-TCS	EGFR ^2^	Hepatocellular carcinoma	Inhibits the growth of solid tumors in nude mice	[120]
2011	EGF-TCSredlk	EGFR	Hepatocellular carcinoma	Anti-cancer activity in vivo and in vitro	[121]

^1^ CEA: carcinoembryonic antigen. ^2^ EGFR: Epidermal Growth Factor Receptor.

## Data Availability

All data are contained within this article.

## Supplementary Materials

The following supporting information can be downloaded at: https://www.mdpi.com/article/10.3390/toxins14030178/s1, Figure S1: Multiple sequence alignment of TCS with other RIPs.
