# Supplementary material for: A Sixty-Year Research and Development of Trichosanthin, a Ribosome-Inactivating Protein
Source: Toxins (Basel). 2022 Feb 27;14(3):178. doi: 10.3390/toxins14030178 (PMC8950148; doi:10.3390/toxins14030178)
Supplement: Supplementary file 1 [file toxins-14-00178-s001.zip › toxins-1612531-supplementary.pdf]

toxins

MDPI

A Sixty-Year Research and Development of Trichosanthin, a Ribosome-Inactivating Protein

Jia-Qi Lu, Kam-Bo Wong and Pang-Chui Shaw

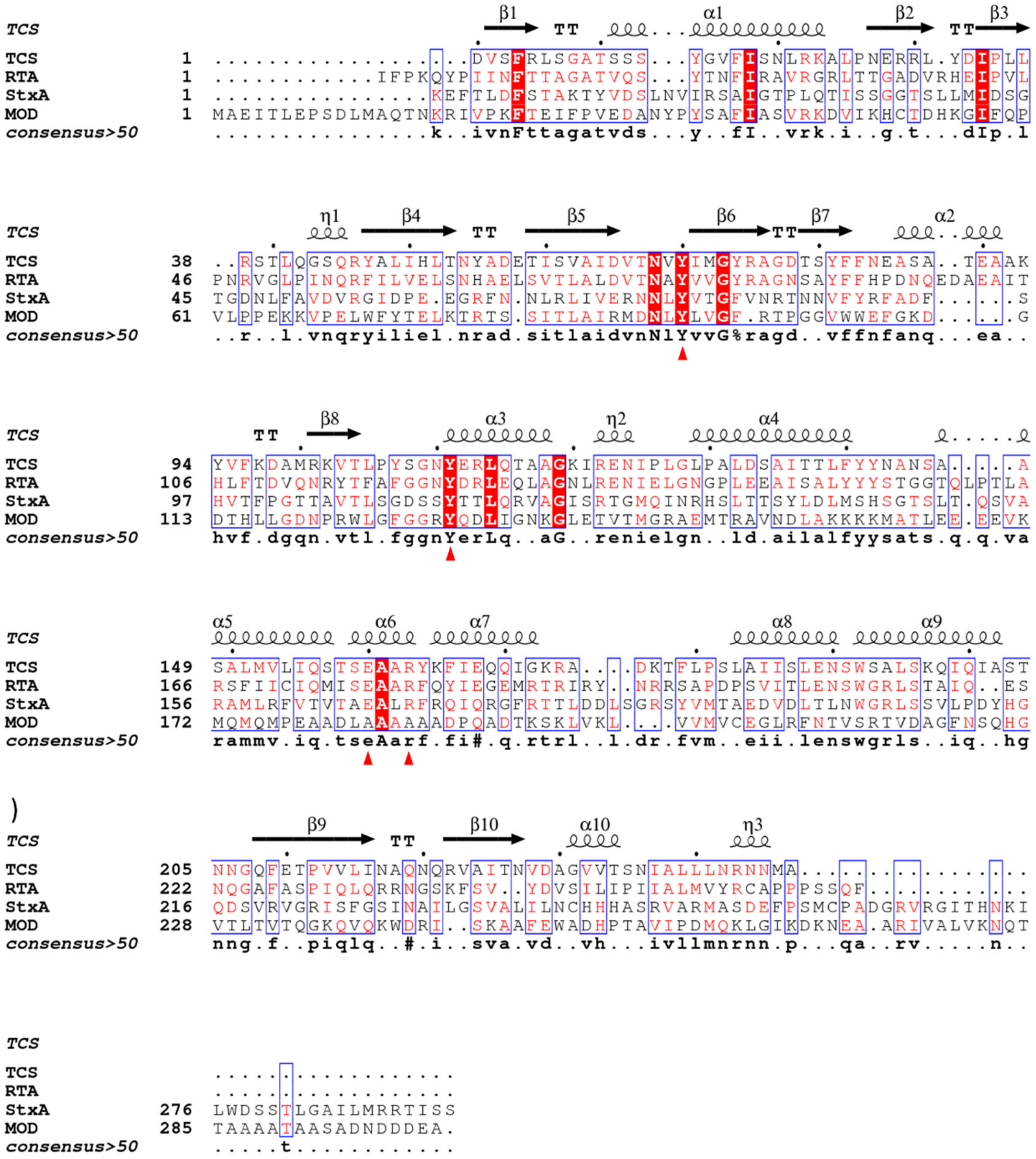

Figure S1. Multiple sequence alignment of TCS with other RIPs. The conserved active sites of RIPs were marked with red triangles. Trichosanthin (TCS, Uniprot accession: Q6BBQ4, PDB code: 2JDL); Ricin A chain (RTA, Uniprot accession: P02879); Shiga toxin subunit A (StxA, Uniprot accession: Q9FBI2); maize ribosome-inactivating protein (MOD, Uniprot accession: P28522)
